# A rare case report of tricorpora penile fracture associated with urethral disruption

**DOI:** 10.1016/j.ijscr.2022.107351

**Published:** 2022-06-25

**Authors:** Gede Wirya Kusuma Duarsa, Muhlis Yusuf, Yudhistira Pradnyan Kloping, Ilham Akbar Rahman, Soetojo

**Affiliations:** a Department of Urology, Faculty of Medicine, Universitas Udayana, Sanglah General Hospital, Bali, Indonesia; b Department of Urology, Faculty of Medicine, Universitas Airlangga, Dr. Soetomo General-Academic Hospital, Surabaya, East Java, Indonesia

**Keywords:** Penile fracture, Urethral disruption, Tricorpora rupture, End-to-end anastomosis urethroplasty, Penile degloving, Sexual health

## Abstract

Penile fracture is defined as a tear of tunica albuginea that covers the corpus cavernosum during an erection. It is a rare finding that both the corpora cavernosum and corpora spongiosum are involved in penile fracture. Herewith, we reported a rare case of 44 years old presented with penile fracture during woman on top sex position with both corpora cavernosum and corpus spongiosum rupture with urethral disruption. On clinical examination, the penis was swollen, and there was a sudden loss of erection and ecchymosis. Cystoscopy examination revealed urethral rupture. Emergent surgical repair was then performed. During emergency surgery, we found a defect of 3 cm in bicorporal cavernosa with urethral and corpus spongiosum disruption. The penis was degloved, and debridement with water-tight suturing of tunica albuginea was performed to repair the tear in corpora cavernosa. End-to-end anastomosis urethroplasty with spatulation was also performed to repair the urethra. After 21 days following surgery, erectile function was good and no difficulties in voiding function as shown in uroflowmetry result with Qmax >15 mL/s. The patient had a favorable recovery. This was a rare case report, and with early and prompt surgical intervention, this case could result in a good outcome in preserving erectile function and voiding function.

## Highlights

• Penile fracture is an emergency condition in field of urology
• The presence of tricorporal fracture involving both corpora cavernosa and corpora spongiosum was considered rare case
• The diagnosis of penile fracture was based on clinical presentation of history taking and physical examination
• Immediate surgical treatment involving penile degloving and surgical repair resulted in promising outcome

## 1 Introduction and importance

Penile fracture is the condition of surgical emergency that occurs due to trauma to the erect penis [1]. Most of the patients did not seek medical treatment due to embarrassment; therefore, most of cases were in delayed treatment [2]. Penile trauma occurs as a result of tears in tunica albuginea, corpora cavernosa, or corpos spongiosum [3]. Sexual intercourse was the most common cause of trauma in the erect penis. When the penis slips out of the vagina during vigorous sexual intercourse, the penis may hit the symphysis pubis or perineum, resulting in penile fracture [1]. Clinical manifestations most commonly were a cracking or popping sound followed by immediate detumescence. The penis could also show signs of rapid swelling, extensive ecchymosis, severe pain, and marked deformity [4]. History taking, physical examination and imaging are the modalities that can be used in the diagnosis of this condition. Early detection and early surgical treatment are essential for optimal outcome [5]. The tears in corpora cavernosa are most commonly unilateral. Therefore, it was a rare finding if both corpora cavernosa were involved in a penile fracture, found in only around 5–14 % of cases [6]. Furthermore, the incidence of urethral rupture has been reported at approximately 10–20 % in all penile fracture cases [7]. Combined with these two, the incidence of both bilateral corpora cavernosa rupture, and corpus spongiosum rupture associated with urethral disruption was only 9–20 % from all penile fracture cases [8]. This work has been reported in line with the SCARE and PROCESS criteria [9], [10]. We report one of the rare cases of penile fracture involving the tricorpora which are both corpora cavernosa, and corpus spongiosum with urethral disruption.

## 2 Case presentation

**Fig. 1:**
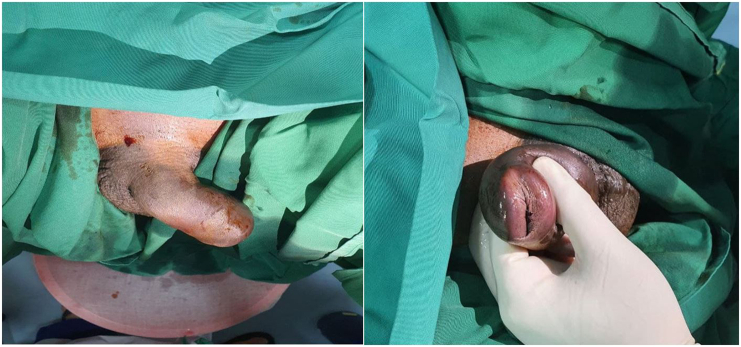
Clinical signs of penile fracture.

A 44 years old male presented to an emergency department with a swollen penis since 3 h ago. The penis was swollen after the patient had vigorous sexual intercourse with his wife so-called “woman on top” sex position. There was a crack sound followed by swelling afterward. He suddenly felt severe pain, followed by immediate loss of erection. Blood was discharged from the tip of his penis. There was no previous history of diabetes mellitus, hypertension, or previous history of surgery. He denied any sexual disorder and history of PDE-5 inhibitor use. On physical examination, his penis was swollen showing the signs of ecchymosis (Fig. 1). Urethral bleeding was visualized. He had voiding difficulties which highly suggested urethral injury. Laboratory finding was normal.

**Fig. 2:**
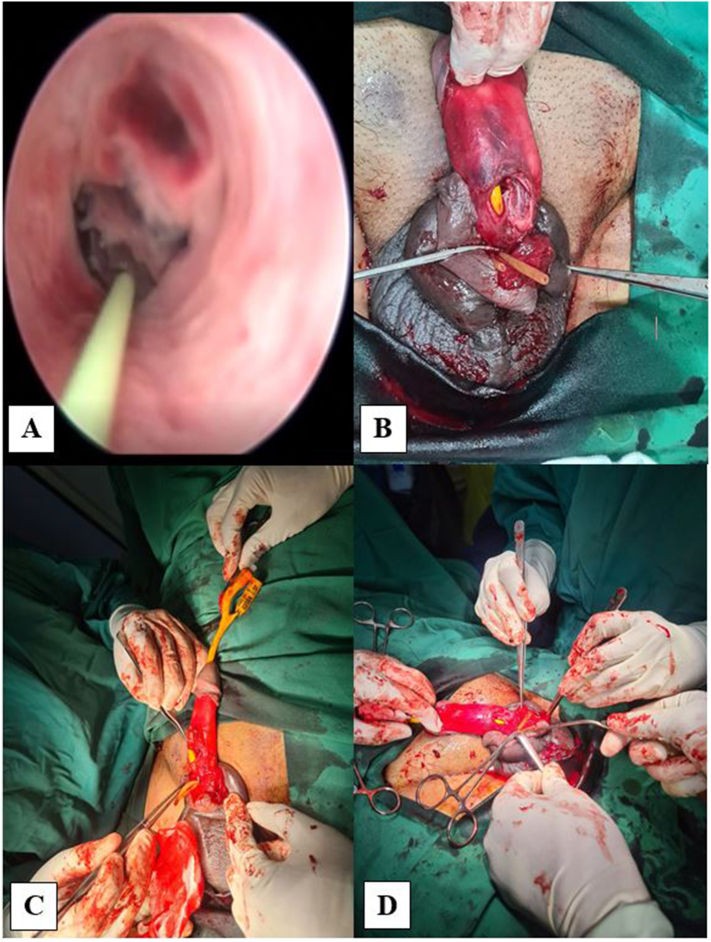
Intraoperative finding. (A) Cystoscopy examination showing urethral disruption, (B) defect of 3 cm in bicorpora cavernosa and spongiosa, (C–D) urethral and corpus repair.

The patient was immediately scheduled for an operative procedure. Suspected urethral disruption with its localization was evaluated prior to surgical repair using cystoscopy, and eventually revealed urethral rupture (Fig. 2A). An incision was performed in the coronal sulcus to deglove the penis. The rupture of tunica albuginea was found extending into corpora cavernosa (Fig. 2B). End-to-end anastomosis was performed in the urethra using vicryl 4–0 with interrupted sutures. Debridement with water-tight suturing of the tunica albuginea was performed to repair the tear in corpora cavernosa. Erection test was then performed with the result showing that there was no leakage. Penile reconstruction was performed with skin suturing to the glans penis. After the procedure, the penis was covered with a bandage. The intraoperative finding was presented in Fig. 2.

**Fig. 3:**
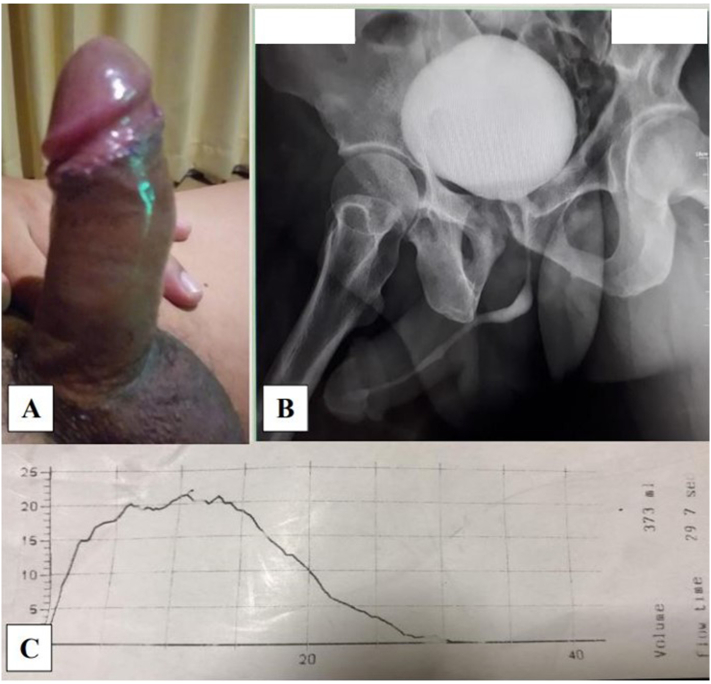
Post-operative day 21 following surgery (A) Erectile function of the penis with EHS score 4, (B) urethrography, and (C) uroflometry test with the result of 22.

An uneventful post-operative period following surgery was found. After 21 days of the follow-up period, uroflowmetry and urethrography examination revealed a normal result. Erectile function was normal with a normal erection hardness score of 4. No deformity in penile curvature was seen, and no difficulty in voiding function was revealed, as shown in the uroflowmetry result with Qmax >15 mL/s. The patient did not complain of any pain during sexual intercourse. Fig. 3 shows the post-operative follow-up 21 days after surgery.

## 3 Clinical discussion

This is a rare case of penile fracture involving the tricorpora which are both corpora cavernosa, and corpus spongiosum with urethral disruption. This case report demonstrated appropriate management with a rare presentation of penile fracture involving the tricorpora. The penis is consisted of erectile tissue, which was arranged in columnar order. There were two dorsolateral corpora cavernosa and one ventral corpora spongiosum, each was coated by the tunica albuginea. The urethra runs through along the corpora spongiosum and forms the glans penis at its tip. The ventral corpora spongiosum was coated by Buck's fascia [3], [4]. Trauma to the flaccid penis does not result in any damage because the location is protected. The tunica albuginea possesses a thickness of 2 mm. However if the penis is in the erect state, the penis is filled with blood, and the thickness of tunica albuginea reduces to 0.25–0.5 mm. This leads to susceptibility to traumatic injury [3]. Several activities were reported to cause the penile fracture, including vigorous sexual intercourse, a blow to the penis, and dangerous manipulation to achieve tumescence [3]. Various sexual positions often cause injury to the penis. The most common vigorous sexual position was doggy style. A previous study reported that doggy style accounted for double fractures in 67 patients (10 %). Another study also reported that penile fracture usually occurs if the woman is in a superior position. It is due to the entire body weight lands on the erect penis or when the erect penis hits the woman's perineum [1].

The diagnosis of penile fracture was based on history taking and physical examination, in which 90 % of the symptoms were sudden cracking or snapping sound, immediate pain, and a significant subcutaneous hematoma [11]. Blood discharge from the meatus and voiding difficulty should raise awareness of urethral injury. In relation to our case, the patient experienced a snapping sound during vigorous sexual intercourse [12]. They performed “woman on top” sexual position during their sexual intercourse. Vigorous sexual intercourse was one of the main causes of penile fracture. High energy trauma during this sexual position injured the corpora cavernosa and caused urethral disruption. A previous study reported that urethral rupture only accounts for 3 % of penile trauma [13]. Most commonly tear of the tunica albuginea was unilateral. However, bilateral ruptures can occur in 5–14 % of cases [6]. In relation to our case, our patient had both corpora cavernosa injuries with urethral disruption. The possible explanation of urethral disruption with both corporal tears was the extensions of a ventral tear along the midline to involve the corpus spongiosum.

Retrograde urethrography (RUG) should be considered in this case because it is inexpensive, easy to perform, and highly accurate [14]. However, urethrography is of low yield if the patient has no hematuria, blood at the meatus, and no voiding symptoms. The diagnosis of a penile fracture can be through only the clinical presentation. However, in several unclear/atypical cases, ultrasound (US) and MRI can be used to detect disruption of tunica albuginea [15]. The use of cavernosography could also be useful in acute fracture of the penis in the hope to evaluate the extent of injury in corpus cavernosum depending in certain circumstances which the exact nature of the damage is not clear clinically [16]. However, in our case, the clinical picture was apparent. The condition was emergent suggesting urethral rupture as shown with the presence of urinary retention. Therefore, we performed an endoscopic cystoscopy procedure to directly evaluate the urethra. Cystoscopy performed at the time of exploration is the most sensitive means to assess for urethral injury. It is in accordance with the report from Ciamack Kamdar, et al. in 2008, which suggested that cystoscopy in the operating room is recommended instead of a retrograde urethrogram if a urethral injury is suspected [17]. Consequently, intraoperative cystoscopy is an appropriate method of urethral evaluation for this patient.

Compared to conservative treatment, surgical treatment has produced better outcome and shorter hospital stays with less complication rate. Surgical intervention with the closure of the tunica albuginea can ensure the lowest rate of negative long-term sequelae and has a favorable effect on the psychological wellbeing of the patient. A previous study reported the comparison of surgical and conservative management with 50 % and 4 %, respectively [14]. Related to our case, debridement with the closure of the tunica albuginea using water-tight suturing was performed to repair the tear in corpora cavernosa. A circumferential incision was performed in the coronal sulcus, which enables complete degloving of the penis.

The importance of penile degloving, in this case, was for a comprehensive search to check any missed injuries [18]. This technique was also described previously in which circumferential incision proximal to the coronal sulcus establishes complete degloving of the penis [19]. Urgent surgical intervention, in this case, was undertaken to repair the corporal tears and urethral injury. This was approved by the previous study, which suggested that surgical treatment for penile fracture and urethral injury should be taken as soon as possible to produce fewer complications and optimize outcomes [6], [20].

## 4 Conclusion

Penile fracture is one of the emergency conditions in urology. Bilateral rupture of corpora cavernosa with urethral disruption is an uncommon case. The diagnosis of penile fracture was based on clinical presentation. The classic symptoms were snapping or cracking sounds, followed by immediate detumescence and severe pain. Prompt surgical treatment involving penile degloving and surgical repair resulted in a promising outcome.

## Ethical approval

Ethical approval has been acquired in this study.

## Sources of funding

No funding was granted in this study.

## Informed consent

Written informed consent was obtained from the patient for publication of this case report and accompanying images. A copy of the written consent is available for review by the Editor-in-Chief of this journal on request.

## Author contributions

Conceptualization – GWK, MY, IAR; Data curation – GWK, MY; STJ; Materials – GWK, MY; Formal Analysis – GWK, MY, IAR, STJ; Investigation – GWK, MY, IAR; Methodology – MY, IAR; Supervision – GWK; Writing original draft – GWK, MY, IAR; Writing, review and editing – GWK, STJ.

## Registration of research studies

Not applicable.

## Guarantor

Gede Wirya Kusuma Duarsa.

## Declaration of competing interest

No conflict of interest in this study.
